# Elucidating the Antimycobacterial Mechanism of Action of Decoquinate Derivative RMB041 Using Metabolomics

**DOI:** 10.3390/antibiotics10060693

**Published:** 2021-06-10

**Authors:** Kirsten E. Knoll, Zander Lindeque, Adetomiwa A. Adeniji, Carel B. Oosthuizen, Namrita Lall, Du Toit Loots

**Affiliations:** 1 Human Metabolomics, North-West University, Private Bag x6001, Box 269, Potchefstroom 2531, South Africa; 25773267@student.g.nwu.ac.za (K.E.K.); zander.lindeque@nwu.ac.za (Z.L.); 26352842@student.g.nwu.ac.za (A.A.A.); 2 Department of Plant and Soil Sciences, Faculty of Natural and Agricultural Sciences, University of Pretoria, Pretoria 0002, South Africa; carel.oosthuizen@uct.ac.za (C.B.O.); namrita.lall@up.ac.za (N.L.); 3 School of Natural Resources, University of Missouri, Columbia, MO 65211, USA

**Keywords:** decoquinate derivative RMB041, *Mycobacterium tuberculosis*, tuberculosis, metabolomics, GCxGC-TOFMS, mechanism of action

## Abstract

Tuberculosis (TB), caused by *Mycobacterium tuberculosis* (*Mtb*), still remains one of the leading causes of death from a single infectious agent worldwide. The high prevalence of this disease is mostly ascribed to the rapid development of drug resistance to the current anti-TB drugs, exacerbated by lack of patient adherence due to drug toxicity. The aforementioned highlights the urgent need for new anti-TB compounds with different antimycobacterial mechanisms of action to those currently being used. An *N*-alkyl quinolone; decoquinate derivative RMB041, has recently shown promising antimicrobial activity against *Mtb*, while also exhibiting low cytotoxicity and excellent pharmacokinetic characteristics. Its exact mechanism of action, however, is still unknown. Considering this, we used GCxGC-TOFMS and well described metabolomic approaches to analyze and compare the metabolic alterations of *Mtb* treated with decoquinate derivative RMB041 by comparison to non-treated *Mtb* controls. The most significantly altered pathways in *Mtb* treated with this drug include fatty acid metabolism, amino acid metabolism, glycerol metabolism, and the urea cycle. These changes support previous findings suggesting this drug acts primarily on the cell wall and secondarily on the DNA metabolism of *Mtb*. Additionally, we identified metabolic changes suggesting inhibition of protein synthesis and a state of dormancy.

## 1. Introduction

Tuberculosis (TB), caused by *Mycobacterium tuberculosis* (*Mtb*)*,* is currently considered the deadliest infectious disease worldwide [1]. TB causes about 1.3 million deaths annually, in addition to approximately 300,000 deaths of patients coinfected with the human immunodeficiency virus (HIV), while newly infecting 10 million people per annum [1,2]. Furthermore, the prevalence of multiple-drug resistant (MDR) and extensively drug resistant (XDR) TB is also on the rise [3,4]. The urgency for developing new anti-TB drugs that are less expensive, less cytotoxic, and more efficient, as well as being readily co-administered with HIV treatment, is indisputable. Currently, the first-line treatment for TB entails a six-month “directly observed treatment short-course” (DOTS), which includes using isoniazid (INH), ethambutol (EMB), pyrazinamide (PZA), and rifampicin (RIF) in combination [5]. Infection with MDR- and XDR-TB requires more expensive treatment approaches, for a longer treatment duration, using drugs that have a higher risk of adverse side effects [6,7], which in turn leads to poorer patient compliance and subsequently an escalated increase of drug resistant TB [8]. Further contributing factors to the development of drug resistant TB include inaccurate diagnosis, unsupervised treatment protocols, and poor economic status [9,10,11]^,^, and in 2020, this was further exacerbated by the COVID-19 pandemic [1,12]. Over the last 50 years, the only new antimycobaterial compounds to be approved, as last-resort options, are the anti-TB drugs linezolid, bedaquiline, and delamanid [3,4,5,13]. Recently, the WHO and the UN held its first high-level meeting aimed at initiating an urgent global response to end the TB epidemic by 2030 [1]. However, certain objectives need to be met before this goal can be achieved. Most importantly, elucidating the antimycobacterial mechanisms of action of drugs is considered essential and a means towards finding novel *Mtb* drug compounds and targets [14]. Furthermore, the mechanisms of drug resistance also need to be fully understood [15]. Equally important is economic viability, particularly in high-burden countries with insufficient funding for extremely long, yet mandatory, clinical trials. It is thus beneficial to further investigate already approved drugs with established pharmacokinetic properties and safety profiles [16].

Decoquinate (DQ), an anticoccidial quinolone used as a broad-spectrum antibiotic, has gained interest for its efficacy against malaria, toxoplasmosis, and tuberculosis [17], accompanied by an excellent safety profile [18], high permeability into the cells, and its relatively low costs [19]. Recently, an *N*-alkyl DQ derivative (RMB041) showed high activity against *Mtb* (MIC_90_ = 1.61 µM), with similar in vitro potency to that of ciprofloxacin (1.5–12 µM), gatifloxacin (0.66–1.3 µM), and moxifloxacin (0.62–1.3 µM) [20]. Its permeability (LogP_app_ = −4.8, where compounds with values > −5 are considered highly permeable [21]) confers a benefit for drug diffusion through the lipid cell wall [22] and penetration of infected macrophages and granulomatous lesions in the lungs [23,24]. This drug also shows low cytotoxicity in human fetal lung fibroblasts [25], as well as promising pharmacokinetic properties, including an intravenous elimination half-life (t_1/2_) of 62.3 h in murine models and a low human intrinsic clearance rate (CL_int_) (16 µL/min/mg) [2]. The t_1/2_ is relatively long when compared to that of other well-known anti-TB drugs such as RIF (7.19 h) [26,27], EMB (3 h) [28], INH (1.7 h) [29], and PZA (1.05 h) [30], whereas the CL_int_ performs well compared to that of EMB (>75 µL/min/mg) and INH (<22 µL/min/mg) but less so in comparison to RIF (<10 µL/min/mg) [31]. Drugs with lower CL_int_ and longer t_1/2_ require less frequent administration/dosing, which could improve patience compliance and contribute to lowering the prevalence of drug resistance [32]. Furthermore, DQ derivative RMB041 could hold the key to shortening the duration of TB treatment and reduce the current costs of therapy [33]. Considering this, DQ derivative RMB041 is a promising anti-TB candidate. However, little is known about its antimycobacterial mechanism of action. Contrary to DQ, which primarily targets the ubiquinol-binding pocket, the RMB041 derivative rather shows activity against the cell wall and DNA metabolism of *Mtb* as its primary and secondary targets, respectively [25]. The exact underlying mechanism of action, however, is yet to be determined.

In this study, a two-dimensional gas chromatography coupled with time-of-flight mass spectrometry (GCxGC-TOFMS) metabolomics approach, combined with universally connected metabolic libraries and advanced statistical analysis, was used to identify the metabolite markers best differentiating *Mtb* treated with and without DQ derivative RMB041.

## 2. Results

### 2.1. Data Overview

**Figure 1:**
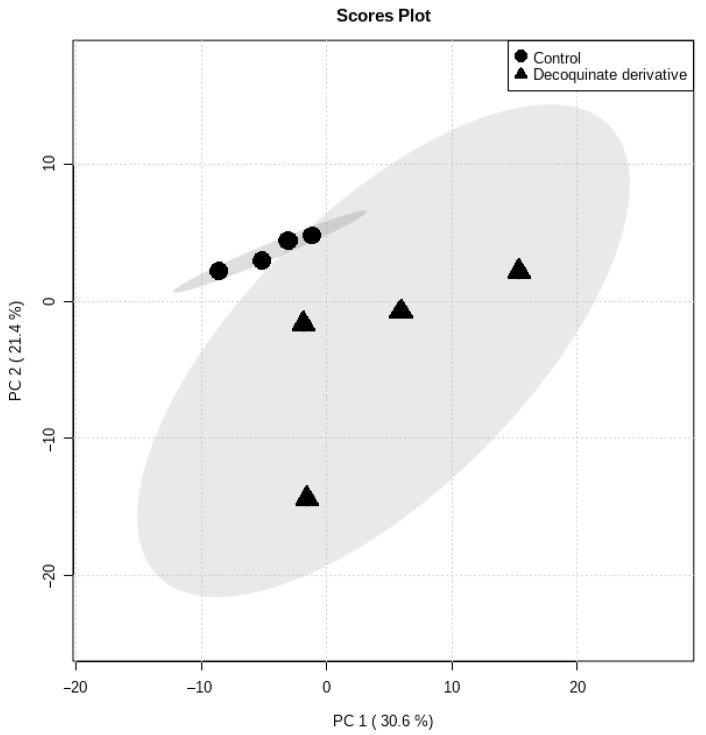
PCA scores plot obtained from GCxGC-TOFMS whole metabolome analysis of *Mtb* samples in the presence and absence of DQ derivative RMB041.

Principal component analysis (PCA) was initially used to get an overview of the natural grouping of metabolic data (Figure 1). The total variance described by the first two principal components (PCs) was 52%, of which PC1 accounted for 30.6% and PC2 for 21.4%, respectively. The PCA scores plot of the metabolite data analyzed by GCxGC-TOFMS shows clear clustering between *Mtb* treated with DQ derivative RMB041 and those cultures that were not (Figure 1). The scores plot also shows that the variance of the treated *Mtb* group is clearly greater than the controls, as a result of greater variance in the metabolite concentrations.

### 2.2. Marker Selection

**Figure 2:**
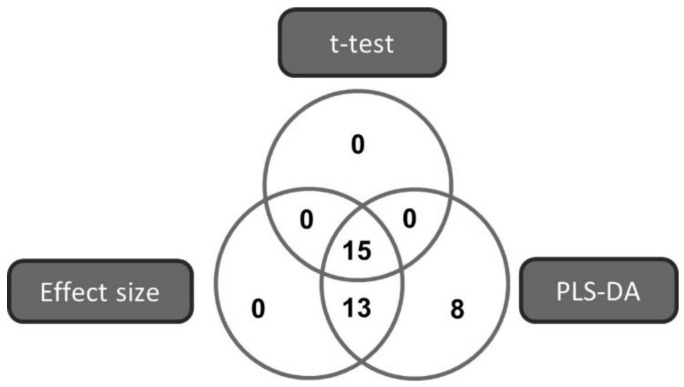
Venn diagram illustrating the multi-statistical approach for selecting the metabolites that best describe the variation detected in the metabolome of *Mtb* cultured with and without DQ derivative RMB041.

Based on compliance to the following criteria: a PLS-DA VIP value > 1 [34], a *t*-test *p*-value < 0.05 [35] or an effect size > 0.8 [36], 36 metabolites were selected as markers best describing the difference between *Mtb* treated with DQ derivative RMB041 and untreated *Mtb* control samples (Figure 2). The metabolites are listed in Table 1, according to their VIP values.

## 3. Discussion

**Figure 3:**
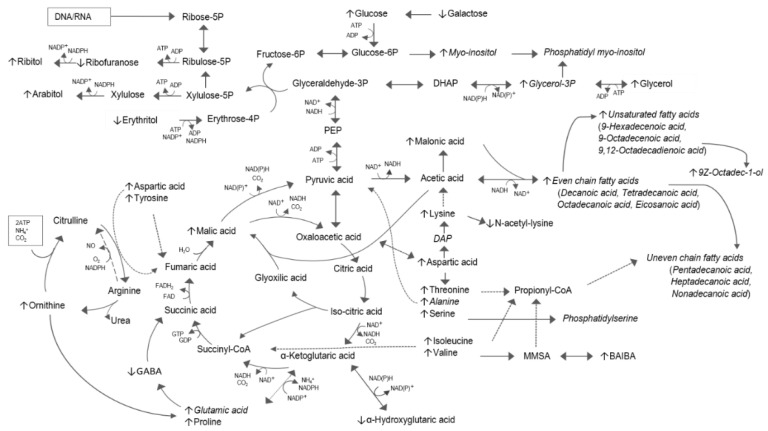
Metabolomic pathway map of DQ derivative RMB041-treated *Mtb*. The metabolite markers best describing the variation in the metabolome compared to that of untreated *Mtb* are represented with up or down arrows, indicating elevated or reduced concentrations, respectively. The dotted lines represent flux of amino acids from nucleotide degradation into the central carbon metabolism. Metabolites that are incorporated into the cell wall are indicated in italic.

In this study we identified a number of significantly altered metabolites induced by the administration of DQ derivative RMB041 to *Mtb* culture. In the light of known metabolism and previous DQ derivative RMB041 findings, the interpretation of these selected metabolite markers, and their associated metabolic pathways, lead to better elucidation of the antimycobacterial mechanism of action of this drug. The most prominently altered pathways (amino acid metabolism, fatty acid metabolism, pentose phosphate pathway (PPP), and the urea cycle) are mapped in Figure 3.

Preeminent in the *Mtb* treated with DQ derivative RMB041 is the elevation of many of the even and odd saturated fatty acids of between 10 to 20 carbons (C10:0-C20:0). This was also true for the Δ^9^-mono-unsaturated fatty acids (Δ^9^ C18:19), as previously mentioned, and 9-hexadecenoic (Δ^9^C16:1), in addition to the Δ^9,12^-poly-unsaturated fatty acid 9,12-octadecenoic (Δ^9,12^ C18:2). These indicate a strong upregulated synthesis towards cell wall repair [37] and an accumulation thereof due to DQ derivative RMB041 inhibiting this process, which strengthens previous findings, suggesting the antimycobacterial activity of DQ derivative RMB041 targeting the cell wall [25]. The distinctly multilaminate cell wall of *Mtb* consists of a peptidoglycan (PG) layer covalently attached to arabinogalactan (AG) [38], which itself attaches to mycolic acids. Interspersed is the mycobacterial plasma membrane, consisting of glycerophospholipids and glycerolipids, phosphatidyl myo-inositol mannosides (PIM), lipomannans (LM), and lipoarabinomannans (LAM) [39], all of which are considered critical for maintaining cell wall integrity [40], and PIM, which contributes to the low permeability of the cell envelope and intrinsic tolerance to antibiotics [41]. The synthesis of PIM requires myo-inositol and glycerol-3-phosphate (glycerol-3P), which were also elevated in this study. Myo-inositol is produced via the conversion of glucose-6-phosphate (glucose-6P) to myo-inositol 1-phosphate prior to dephosphorylation by myo-inositol monophosphate phosphatase [42]. Glycerol-3P is acetylated to produce phosphatidate [43], which serves as the precursor for all glycerolipids [44]. The saturated fatty acids identified in this study are synthesized by fatty acid synthase type I (FAS I) and provide fatty acyl-coenzyme A’s (CoA) to FAS II for elongation [45]. FAS I and FAS II provide acyl-groups for the synthesis of all cell wall components except for AG [38,46,47]. C14:0, C16:0, C18:0, Δ^9^ C16:1, and Δ^9^ C18:1 are considered major fatty acids of the glycerolipids and mycolic acids [48,49,50]. C16:0 and C18:0 are oxidized, in the presence of Fe^2+^, a flavin, NADPH, and O_2_, to produce Δ^9^ C16:1 and Δ^9^ C18:1, respectively [51,52].

The mycobacterial cell envelope also plays an important role during survival of dormant, non-replicating *Mtb* [53,54]. Mycobacterial membrane proteins recognize stress conditions [55] and release acyl-CoA’s for rearrangement of the cell wall components [56] and/or energy metabolism [54]. The expression of the stress response operon requires continuous energy [57], which is mostly derived from NADH and NADPH released during fatty acid metabolism [58]. Furthermore, an upregulated glyoxylate shunt, as opposed to the oxidative half of the tricarboxylic acid (TCA) cycle, along with reduced glycolysis and altered glycerol metabolism, is associated with an *Mtb* shift towards a state of dormancy [59,60,61]. In our investigation, the elevated levels of malic acid, glucose, sugar alcohols (with the exception of erythritol), glycerol-3P, and glycerol (Figure 3) suggest that the same stringent response to dormancy occurs in *Mtb* treated with the DQ derivative RMB041. The elevated levels of ribitol and arabitol in the PPP indicate a metabolic flux of glucose metabolism towards the PPP, and not towards energy production via glycolysis [62]. Furthermore, the decreased concentrations of erythritol confirms the flux towards PPP, and also the aforementioned PIM synthesis via fructose-6-phosphate (fructose-6P) (Figure 3). From the results it is evident that fatty acids are most likely the preferred energy source, further confirmed by the elevated levels of short chain fatty acids (C10:0-C14:0) and malonic acid, which are derived via β-oxidation of FAS I and FAS II products, in the presence of NAD^+^ [63], in the DQ derivative RMB041 treated *Mtb*. Regeneration of NAD^+^ is maintained by the increased expression of isocitrate lyase, leading to enhanced flux through the glyoxylate shunt [64]. This also reduces the release of CO_2_, resulting in a direct carbon flux towards FAS I and FAS II [65], for subsequent energy production [54] and cell wall remodeling [56]. In the presence of ATP, the resultant CO_2_, together with NH_4_ released during amino acid metabolism, are incorporated into the urea cycle via ornithine (which is elevated in the RMB041 treated *Mtb*, indicating an upregulated urea cycle) (Figure 3) [66]. Ornithine serves as the precursor of polyamines, which have previously been associated with antibiotic-induced mutations [67]. Furthermore, ornithine contributes to the reclamation of carbon to the TCA cycle via proline and glutamic acid. This can be obtained via γ-aminobutyric acid (GABA) or via α-ketoglutaric acid (Figure 3) [68]. Notably, DQ derivative RMB041-treated *Mtb* prefers the latter, which could be explained by the necessity of α-ketoglutaric acid to incorporate amino acids, such as aspartic acid, into the TCA cycle [69]. Moreover, NAD(P)H and α-ketoglutaric acid are converted to NAD(P)^+^ and α-hydroxyglutaric acid [70], which was also found to be elevated in this study. This might further indicate an imbalanced NAD(P)^+^/NAD(P)H ratio, supporting the need for NAD(P)^+^ for survival of fatty acids as the main energy source, and/or disruption of the membrane, leading to a disturbed electron transport chain (ETC) and, subsequently accumulation of NADH [71].

The *Mtb* non-replicative phase is also associated with a reduction/stagnation of DNA, RNA, and protein synthesis [72,73]. The accumulation of urea (0.012 vs. 0.002 mg/g; *p* = 0.63) in the *Mtb* treated with DQ derivative RMB041, although not selected as a metabolite marker through the statistical selection process described above, indicates the conservation of nitrogen from protein synthesis [74]. Inhibition of protein synthesis would subsequently disrupt the functionality of membrane proteins and result in an accumulation of fatty acids as a result of inhibition of cell wall synthesis [75]. Urea could also serve as an opposing force to prevent osmotic lysis [76] brought about by dehydration as a result of an altered cell envelope integrity. Aspartic acid, along with ten other amino acids (Table 1), were found elevated in this study. When considering this in light of the elevated levels of sugar alcohols detected in the DQ derivative RMB041 treated *Mtb* group, degradation of the nucleic acids to purine and pyrimidines is evident [77,78]. Speculatively, the direct activity of DQ derivative RMB041 on DNA would most likely result in an attempt towards DNA repair, as was previously indicated by reduced sugar alcohols in *Mtb* treated with ciprofloxacin [79]. In this case, however, we saw an accumulation of both amino acids and sugar alcohols, suggesting DNA replication has not yet been inhibited or repair mechanisms have not yet been activated at the time the cells where harvested. This may, however, occur at a later stage, or at higher DQ derivative RMB041 concentrations perhaps, but in the present study, the *Mtb* treated with DQ derivative RMB041 are most likely still in a non-replicative state after the irreversible cell envelope damage, which is the primary target of DQ derivative RMB041 [25]. This is also supported by previous findings suggesting DNA replication as a secondary target and cell wall metabolism as a primary target of DQ derivative RMB041 [25].

In a study by Rizvi, et al. [80], oxidative stress, inherent with the shift to a dormant state in *Mtb*, resulted in the statistically significant accumulation of the same six (aspartic acid, valine, threonine, alanine, isoleucine, and lysine) of the ten amino acids identified as metabolite markers in the DQ derivative RMB041 treated *Mtb* in our study. The flux of lysine towards acetic acid, and subsequent fatty acid synthesis, is supported by decreased levels of its break-down product, N-acetyl-lysine [81]. Furthermore, the branched chain amino acids, threonine, isoleucine and valine, serve as precursors of propionyl-CoA [82] for elongation of odd chain fatty acids [83]. Flux towards propionyl-CoA from valine is furthermore supported by the increased levels of β-aminoisobutanoic acid (BAIBA) in the DQ derivative RMB041 treated *Mtb*, whereby the intermediate methyl-malonyl semialdehyde (MMSA) is the direct precursor of propionyl-CoA [84].

Serine, one of the elevated amino acid markers in the DQ derivative RMB041 treated *Mtb* group, also serves as a precursor of phosphatidylserine [85]. Translocation of phosphatidylserine on the outer membrane of *Mtb* has been associated with cell death induced by reactive oxygen species (ROS) [86]. ROS are released during the antibiotic-induced stress response and accumulate when DNA repair is unsuccessful, which in turn results in the aforementioned dormant phase [87,88]. Altered PG metabolism, as is brought about by cell wall active antibiotics, has also been linked to changes of growth and division of bacterial cells [89]. This further suggests irreversible inhibition by DQ derivative RMB041 to the cell envelope, which would result in a continuous activation of the stress response operon, requiring non-stop energy, supplied by fatty acid metabolism, and, partially, the TCA cycle, followed by the induction of a dormant state, whereby cell growth and division is ultimately inhibited.

In summary, this metabolomics approach better characterized the antimycobacterial mechanism of decoquinate RMB041, which not only confirmed previously propose mechanisms of action but also suggests possible new mechanisms, which could be further investigated and confirmed using alternative strategies. A possible limitation, however, is the degradation of thermolabile metabolites into smaller unidentifiable “unknown” compounds [90].

## 4. Materials and Methods

### 4.1. Cell Culture

The reagents used for this investigation were purchased from Sigma–Aldrich, St. Louis, MO, USA, unless specified otherwise. The microtiter Alamar Blue assay was used to determine the antimycobacterial sub-minimum inhibitory concentration (IC_50_) of DQ derivative RMB041 [91]. Cell cultures were prepared as delineated by Van Breda et al. [92], with minor modifications. Briefly, *Mtb* H37Rv ATCC 27294, obtained from the Medical Research Council (Pretoria, Gauteng, South Africa), was cultured and maintained on Lowenstein-Jensen (LJ) slants. After four weeks of incubation, a bacterial inoculum of McFarland 1 (approximately 3 × 10^8^ colony-forming units/mL) was prepared in Middlebrook 7H9 broth containing 10% OADC (oleic acid, albumin, dextrose, catalase) (Becton, Dickinson, UK) and 2% PANTA (polymyxin B, amphotericin B, nalidixic acid, trimethoprim, and azlocillin) (Becton, Dickinson, UK). The latter shows a negligible impact on *Mtb* growth and was added to prevent contamination [93]. In five of the ten aliquots, 4 mL DQ derivative RMB041 was dissolved in DMSO (150 µM) and further diluted in Middlebrook 7H9 broth to yield a final concentration of 10 µM (0.2% DMSO). A volume of 1 mL of the prepared inoculum was added to reach a final assay volume of 5 mL, with a bacterial test concentration of 6 × 10 ^7^ CFU/mL DQ derivative RMB041. The untreated *Mtb* control samples were prepared in the remaining five aliquots by adding the bacterial inoculum, as described above, in 4 mL of Middlebrook 7H9 broth (0.2% DMSO). After 5 days of incubation at 37 °C, the mycobacteria cultures were pelleted by centrifuging the samples at 4500 rpm for 15 min. These were then washed with 1 mL of PBS and re-pelleted, as described above. Lastly, the PBS was carefully aspirated, and the samples and the samples were stored at −80 °C until further testing.

### 4.2. Whole Metabolome Extraction Procedure and Derivatization

The extraction and derivatization procedures were carried out as previously described by Beukes et al. [94], with slight modifications. Briefly, 8 mg of each of the ten individually cultured samples were weighed into an Eppendorf tube. As internal standard, 50 µL 3-phenylbutyric acid (0.13125 mg/mL H_2_O) (Sigma–Aldrich, Lot#536478V) was added, and a two-phase extraction was performed with chloroform: methanol: water in a 1:3:1 ration (1 mL in each sample). The Eppendorf tubes, containing a 3 mm carbide tungsten bead in each, were shaken in a vibration mill at 30 Hz for 5 min, followed by centrifugation at 12,000 rpm for 5 min. After transferring each extract to a GC glass vial, the samples were dried under a nitrogen stream. Derivatization was achieved by firstly adding 50 µL methoxamine hydrochloride (Sigma–Aldrich, Lot#BCBP2843V) in pyridine (Lot#S2BC335SV) (at a final concentration of 15 mg/mL) to the glass vials and heating at 50 °C for 90 min, followed by the addition of 40 µL N,O-bis(trimethylsilyl)trifluoroacetamide (BSTFA) with 1% trimethylsilyl chloride (TMSCI) (Lot#BCBW2670) and again heating for 60 min at 50 °C. Lastly, each extract was transferred to a 0.1 mL vial insert, which were placed into their respective GC sample vial prior to injection into the GCxGC-TOFMS.

### 4.3. GCxGC-TOFMS Analysis

The metabolomics analysis was performed using a 4D Pegasus GCxGC-TOFMS (LECO Africa (Pty) Ltd., Johannesburg, South Africa), equipped with a Gerstel Multi-Purpose Sampler (Gerstel GmbH and Co. KG, Mülheim an der Ruhr, Germany) and an Agilent 7890 gas chromatograph (Agilent, Atlanta, GA, USA) coupled to TOFMS (LECO Africa). The GC-MS parameters were set as previously described [94]. A Rxi-5Sil MS capillary column was used as the primary column (28.8 m × 0.25 mm internal diameter, 0.25 µm film thickness, Restec), along with a Rxi-17 capillary column (1.2 m × 0.25 mm internal diameter, 0.25 µm film thickness). The primary GC oven temperature was set at 70 °C for 2 min, and increased at a rate of 4 °C/min to a final temperature of 300 °C, at which it was maintained for an additional 2 min. The secondary oven was set at 85 °C for 2 min, increased at 4.5 °C/min, to a final temperature of 300 °C, at which it was maintained for 4.5 min. Helium, used as a carrier gas, was set to a column flow rate of 1 mL/min and held at a constant temperature of 270 °C. Mass spectrometric data acquisition was carried out using a filament bias of −70 eV, with a 350 s solvent delay, and an acquisition scanned mass range of 50–800 *m*/*z* at 200 spectra/s. To ensure high internal validity, the samples were analyzed in random sequence. The analytical performance of GCxGC-TOFMS throughout the analysis was monitored by adding quality control (QC) samples at the start, in the middle, and at the end of the sequence.

### 4.4. Data Processing, Clean-Up, and Statistics

Mass spectral deconvolution (at a signal to noise ratio of 20), peak alignment, and peak identification was done with ChromaTof software (version 4.32, Leco Corporation, St. Joseph, MI, USA). For the identification of metabolites, their mass fragment patterns were compared to those of compounds in the commercially available database, NIST 11, and an in-house created Organic Acids library, containing previously injected standards. The quality of the data was evaluated by pretreating the data using a standardized metabolomics data clean-up procedure [94]. MS total useful signal was used for data normalization. Considering that the zero values are most likely present in sub-minimum concentrations rather than being completely absent, they were replaced by a value calculated as 20% of the minimum detection limit of the entire dataset [95]. An 80% data filter was then applied to remove compounds that were only detected in two (or less) samples per group [96]. Log transformation and auto-scaling was applied to balance metabolite representations. This prevented domination of compounds with higher concentrations over compounds with lower concentrations [97].

Using MetaboAnalyst (Version 5.0, https:www.metaboanalyst.ca, accessed on 3 May 2021) [98], two multivariate statistic methods were applied: (1) an unsupervised principal component analysis (PCA) and (2) a supervised partial least squares-discriminant analysis (PLS-DA) [99]. Additional univariate statistics were also calculated via (1) *t*-test and (2) effect size values [36].

## 5. Conclusions

DQ derivative RMB041 has been previously proposed to act against *Mtb*, primarily via inhibition/destruction of its cell wall and inhibition/destruction of DNA as a secondary target [25]. This metabolomic investigation indicated an altered metabolite profile (drastically elevated levels of various fatty acids and glycerolipid precursors, amino acids, and urea cycle intermediates) confirming the aforementioned observations in addition to an inhibition of protein synthesis and a state of dormancy. This study not only confirms or improves upon the existing knowledge pool of novel antimycobacterial mechanisms of actions, but also provides a useful tool for investigating repurposed and/or adjunctive drugs against *Mtb*. Furthermore, a better understanding of the dormancy process as indicted here and could lead to new treatment regimens that reduce the emergence of relapse and resistance [100].

## Figures and Tables

**Table 1:** The selected metabolite markers best describing the variance between the metabolome of *Mtb* cultured in the absence (*Mtb* controls) and presence of DQ derivative RMB041.

Metabolite Name (CHEBI ID)	Average Concentration (mg/g Cells) (Standard Deviation)		*t*-Test (*p*-Value)	Effect Size (d-Value)	PLS-DA (VIP)	Fold Change (Log_2_)
	*Mtb* with DQ derivative RMB041	*Mtb* controls				
9-Octadecenoic acid (36021)	0.967 (0.192)	0.560 (0.041)	0.000	34.47	2.59	0.73
Myo-Inositol (17268)	0.024 (0.032)	0.020 (0.002)	0.000	5.298	1.99	0.20
Ribitol (15963)	0.318 (0.513)	0.100 (0.005)	0.006	2.098	1.98	2.18
Arabitol (18403)	0.877 (0.962)	0.790 (0.199)	0.010	2.206	1.98	0.11
Eicosanoic acid (28822)	0.018 (0.025)	0.004 (0.001)	0.000	3.968	1.94	3.50
Erythritol (17113)	0.023 (0.027)	0.028 (0.001)	0.001	3.176	1.92	−0.18
N-Acetyl-Lysine (35704)	0.000 (0.000)	0.004 (0.003)	0.009	1.938	1.81	−1.00
9,12-Octadecadienoic acid (17351)	0.578 (0.827)	0.111 (0.008)	0.016	1.835	1.67	4.21
Valine (16414)	0.039 (0.063)	0.022 (0.005)	0.019	1.908	1.65	0.77
Aspartic acid (17053)	0.018 (0.033)	0.013 (0.004)	0.038	1.341	1.65	0.38
Pentadecanoic acid (42504)	0.017 (0.023)	0.004 (0.001)	0.021	1.648	1.64	3.25
Galactose (28260)	0.004 (0.002)	0.011 (0.001)	0.185	0.747	1.57	−0.64
Glutamic acid (16015)	0.024 (0.043)	0.015 (0.006)	0.032	1.549	1.56	0.60
Lysine (18019)	0.016 (0.030)	0.009 (0.006)	0.047	1.291	1.56	0.78
2-Hydroxyglutaric acid (32796)	0.003 (0.004)	0.002 (0.001)	0.037	1.384	1.53	0.50
Proline (26271)	0.022 (0.041)	0.016 (0.013)	0.060	1.202	1.50	0.38
Glycerol-3-phosphate (15978)	0.012 (0.016)	0.007 (0.002)	0.049	1.308	1.47	0.71
Malonic acid (30794)	0.173 (0.213)	0.039 (0.045)	0.249	0.684	1.46	3.44
Glycerol (17754)	0.994 (1.45)	0.633 (0.031)	0.057	1.171	1.44	0.57
Nonadecanoic acid (NSC11914)	0.499 (0.731)	0.055 (0.009)	0.065	1.127	1.41	8.07
Alanine (16977)	0.321 (0.621)	0.207 (0.152)	0.067	1.156	1.40	0.55
Octadecanoic acid (28842)	0.027 (0.034)	0.005 (0.000)	0.068	1.150	1.40	4.40
β-Aminoisobutanoic acid (33094)	0.035 (0.057)	0.014 (0.004)	0.073	1.310	1.38	1.50
γ-Aminobutyric acid (16865)	0.011 (0.018)	0.016 (0.006)	0.085	1.315	1.34	−0.31
Tyrosine (17895)	0.006 (0.012)	0.003 (0.001)	0.147	0.843	1.32	1.00
9-Hexadecenoic acid (59265)	0.105 (0.153)	0.018 (0.003)	0.099	0.975	1.29	4.83
Heptadecanoic acid (32365)	0.052 (0.069)	0.011 (0.002)	0.103	0.975	1.29	3.73
Ribofuranose (33942)	0.003 (0.003)	0.007 (0.002)	0.239	0.653	1.28	−0.57
Glucose (17243)	0.045 (0.031)	0.037 (0.005)	0.174	0.770	1.24	0.22
Isoleucine (17191)	0.018 (0.032)	0.008 (0.008)	0.216	0.702	1.21	1.25
9Z-Octadec-1-ol (73504)	0.026 (0.036)	0.004 (0.003)	0.276	0.601	1.17	5.50
Decanoic acid (30813)	0.008 (0.009)	0.005 (0.001)	0.157	0.836	1.18	0.60
Malic acid (6650)	0.084 (0.127)	0.054 (0.004)	0.172	0.775	1.11	0.56
Ornithine (15729)	0.029 (0.054)	0.025 (0.015)	0.191	0.841	1.09	0.16
Serine (17822)	0.011 (0.021)	0.004 (0.002)	0.151	0.850	1.06	1.75
Threonine (16857)	0.049 (0.088)	0.016 (0.008)	0.173	0.791	0.93	2.06

## Data Availability

Data sharing is not applicable to this article.
